# Supplementary material for: Nanopore Is Preferable over Illumina for 16S Amplicon Sequencing of the Gut Microbiota When Species-Level Taxonomic Classification, Accurate Estimation of Richness, or Focus on Rare Taxa Is Required
Source: Microorganisms. 2023 Mar 21;11(3):804. doi: 10.3390/microorganisms11030804 (PMC10059749; doi:10.3390/microorganisms11030804)
Supplement: Supplementary file 1 [file microorganisms-11-00804-s001.zip › Illumina_comparison_draft_submission_Microorganisms_supplement.pdf]

Nanopore is preferable over Illumina for 16S amplicon sequencing of the gut microbiota when species-level taxonomic classification, accurate estimation of richness, or focus on rare taxa is required

Márton Szoboszlay[1], Laetitia Schramm[1], David Pinzauti[1], Jeanesse Scerri[1], Anna Sandionigi[2], Manuele Biazzo[1]

[1]The BioArte Ltd, San Gwann, Malta

[2]University of Milan Bicocca, DISCO, Milan, Italy

Supplementary tables

Table S1: Sequencing yield.

Sample	Illumina raw read pairs	Nanopore raw reads	Nanopore trimmed reads	Nanopore quality filtered reads
**Mock**	104889	178513	161733	100506
**Mock**	126117	181006	164518	101523
**Mock**	136677	162365	147664	91350
**A1**	126280	199489	180961	111640
**A1**	138564	198499	179941	111761
**A1**	108634	187606	169693	105160
**B1**	110781	161598	146228	90066
**B1**	130274	177145	159998	99062
**B1**	118184	165345	149378	92191
**AN1**	120367	190866	173056	106591
**AN1**	125680	181985	164611	100666
**AN1**	112429	182352	164744	101844
**MD01**	119083	250784	227059	139562
**MD01**	109324	230994	209913	127798
**MD01**	106072	224344	204196	125022
**S1X**	113130	217891	197947	121585
**S1X**	136979	236344	214118	131186
**S1X**	130658	240542	218454	133814
**S2P**	108602	245526	223887	127347
**S2P**	121263	236612	214531	133116
**S2P**	105438	239594	217607	135519

Table S2: The number of Nanopore reads at each stage of the EzBioCloud MTP pipeline.

Sample	Uploaded	Low quality or not 16S	Not bacterial	Chimeric	Passed	Classified to species, phylotypes, or species groups
**Mock**	100000	1273	0	13431	85296	84551

**Mock**	100001	1293	0	14903	83805	83085
**Mock**	91350	1119	0	12583	77648	76985
**A1**	100000	1615	0	23154	75231	74834
**A1**	100000	1493	0	22369	76138	75776
**A1**	100000	1554	0	23054	75392	75027
**B1**	90066	1799	0	22104	66163	65172
**B1**	99062	2023	0	25646	71393	70284
**B1**	92191	1786	0	22563	67842	66779
**AN1**	100000	1691	0	20953	77356	76633
**AN1**	100000	1717	0	20406	77877	77160
**AN1**	100000	1684	10	20451	77855	77082
**MD01**	100000	1769	5	15234	82992	82217
**MD01**	100000	1952	10	18327	79711	79027
**MD01**	100000	1883	2	16308	81807	81082
**S1X**	100000	2205	0	25069	72726	69749
**S1X**	100000	2176	6	24719	73099	70287
**S1X**	100000	2185	0	24810	73005	70036
**S2P**	100000	1377	0	14416	84207	83562
**S2P**	100000	1473	0	16577	81950	81130
**S2P**	100000	1308	0	13977	84715	83943

Table S3: The number of Illumina reads at each stage of the EzBioCloud MTP pipeline.

Sample	Uploaded	Low quality or not 16S	Not bacterial	Chimeric	Passed	Classified to species, phylotypes, or species groups
**Mock**	100000	825	119	8007	91049	87586
**Mock**	100000	934	165	6860	92041	87598
**Mock**	100000	1094	253	6999	91654	86840
**A1**	100000	527	18	7136	92319	89941
**A1**	100000	486	21	6550	92943	90640
**A1**	100000	507	22	6943	92528	90383
**B1**	100000	526	91	7088	92295	87673
**B1**	100000	686	17	7636	91661	86507
**B1**	100000	505	43	7143	92309	88140
**AN1**	100000	737	55	8143	91065	86083
**AN1**	100000	506	44	8150	91300	86315
**AN1**	100000	690	57	8189	91064	85718
**MD01**	100000	537	2448	8964	88051	82813
**MD01**	100000	545	2054	8823	88578	83297
**MD01**	100000	669	1895	9134	88302	83785
**S1X**	100000	702	180	8651	90467	85917
**S1X**	100000	659	136	8431	90774	85948
**S1X**	100000	671	301	8448	90580	86298
**S2P**	100000	809	108	10299	88784	79804
**S2P**	100000	855	51	8848	90246	80417

**S2P**	100000	787	93	9977	89143	80501

Table S4: Relative abundance (%) of the bacterial genera in the mock community compared to the Nanopore and Illumina results from the EzBioCould MTP pipeline.

Genus	Composition	Nanopore			Illumina		
Akkermansia	0.97	1.61	1.69	1.58	0.67	0.58	0.39
Bacteroides	9.94	14.06	14.27	13.95	11.26	8.73	14.96
Bifidobacterium	8.78	6.75	7.28	6.99	8.24	9.66	8.28
Clostridioides	2.62	5.43	5.06	5.43	1.69	2.70	2.15
Clostridium	0.0002	0	0	0	0.270	0.013	0.023
Enterococcus	0.0009	0	0	0	0.0868	0.0054	0.0011
Escherichia	12.12	10.82	10.97	10.63	5.41	8.42	7.53
Faecalibacterium	17.63	15.00	14.58	14.64	13.70	17.37	17.37
Fusobacterium	7.49	7.64	7.38	7.09	9.09	13.61	4.47
Lactobacillus	9.63	5.68	5.67	6.01	2.34	4.13	3.26
Prevotella	4.98	6.58	6.44	6.48	5.62	0.94	4.14
Roseburia	9.89	7.15	7.11	7.12	5.79	6.78	6.11
Salmonella	0.009	0.018	0.024	0.012	0	0	0
Veillonella	15.87	19.24	19.52	20.06	5.67	3.33	3.99
**Total**	99.93	99.97	99.98	99.99	69.83	76.27	72.67

Table S5: Relative abundance (%) of the bacterial genera in the mock community compared to the Illumina results from the QIIME2 and mothur pipelines.

Genus	Composition	Illumina QIIME2			Illumina mothur		
Akkermansia	0.97	0.71	0.63	0.38	0.68	0.58	0.36
Bacteroides	9.94	11.30	8.93	15.36	9.51	7.85	13.95
Bifidobacterium	8.78	8.22	9.66	8.31	8.23	9.57	8.13
Clostridioides	2.62	1.75	2.75	2.19	1.69	2.60	2.10
Clostridium	0.0002	0.24	0.0060	0.015	0.23	0.012	0.019
Enterococcus	0.0009	0.11	0	0	0.081	0.0037	0.00086
Escherichia	12.12	5.56	8.62	7.74	5.66	8.43	7.58
Faecalibacterium	17.63	13.73	17.86	17.58	14.20	17.74	17.59
Fusobacterium	7.49	9.13	13.80	4.47	9.22	13.60	4.39
Lactobacillus	9.63	2.43	4.25	3.43	2.28	3.10	2.25
Prevotella	4.98	5.67	0.95	4.27	5.66	0.91	4.13
Roseburia	9.89	4.44	5.68	5.01	4.38	5.58	4.91
Salmonella	0.009	0	0	0	0	0	0
Veillonella	15.87	5.64	3.34	4.16	5.68	3.28	4.03
**Total**	99.93	69.0	76.5	72.9	67.6	73.3	69.5

Table S6: The percentage of reads classified into species and genera in the faecal samples.

	Reads classified as species or phylotypes (%)		Reads classified as genera or phylotype genera (%)	
**Sample**	Nanopore	Illumina	Nanopore	Illumina

**A1**	86.2	53.0	99.98	99.40
**A1**	86.2	52.8	99.99	99.44
**A1**	85.6	51.5	99.98	99.51
**AN1**	92.4	28.4	99.97	99.10
**AN1**	92.4	30.8	99.93	99.16
**AN1**	92.0	31.1	99.98	99.27
**B1**	94.8	40.2	99.96	99.35
**B1**	94.5	39.7	99.97	99.02
**B1**	94.7	38.7	99.95	99.33
**MD01**	83.2	53.8	99.77	98.46
**MD01**	82.2	53.0	99.89	99.18
**MD01**	82.1	53.5	99.83	99.06
**S1X**	91.8	53.8	99.79	99.27
**S1X**	92.0	53.1	99.90	99.25
**S1X**	91.9	53.6	99.75	99.34
**S2P**	97.4	52.3	100.00	99.24
**S2P**	96.8	52.5	99.98	98.95
**S2P**	97.3	51.1	100.00	99.12

Table S7: The number of species-level taxa detected in the faecal samples.

	**Species**		**Species groups**		**Phylotypes**		**Phylotype groups**	
**Sample**	Nanopore	Illumina	Nanopore	Illumina	Nanopore	Illumina	Nanopore	Illumina
**A1**	82	214	18	309	46	933	1	115
**A1**	82	198	15	286	51	791	1	101
**A1**	88	193	18	315	49	916	1	107
**AN1**	127	241	32	352	130	967	1	105
**AN1**	117	187	31	298	123	816	1	92
**AN1**	122	229	30	316	122	965	1	116
**B1**	99	213	15	297	164	900	1	102
**B1**	96	245	14	343	158	1065	2	117
**B1**	93	236	11	355	158	864	1	92
**MD01**	95	197	25	258	179	801	1	88
**MD01**	101	195	25	293	189	946	1	109
**MD01**	102	218	24	307	192	1139	2	123
**S1X**	99	240	18	316	234	1158	1	129
**S1X**	104	231	20	324	249	1089	1	110
**S1X**	99	219	20	328	241	1113	2	122
**S2P**	92	242	16	351	104	1124	1	135
**S2P**	87	292	11	394	102	1236	1	128
**S2P**	84	271	9	411	103	1146	1	120

Table S8: The number of genera detected in the faecal samples.

	**Genera**		**Phylotype genera**	
**Sample**	Nanopore	Illumina	Nanopore	Illumina

**A1**	63	392	5	354
**A1**	64	376	6	318
**A1**	68	388	6	378
**AN1**	94	452	31	418
**AN1**	91	360	28	337
**AN1**	89	398	29	391
**B1**	83	395	40	363
**B1**	76	439	45	442
**B1**	77	431	42	374
**MD01**	89	353	56	315
**MD01**	92	392	59	349
**MD01**	90	406	65	435
**S1X**	91	415	65	428
**S1X**	94	411	76	410
**S1X**	93	409	72	439
**S2P**	77	435	26	424
**S2P**	72	477	27	494
**S2P**	71	483	26	469

Supplementary figures

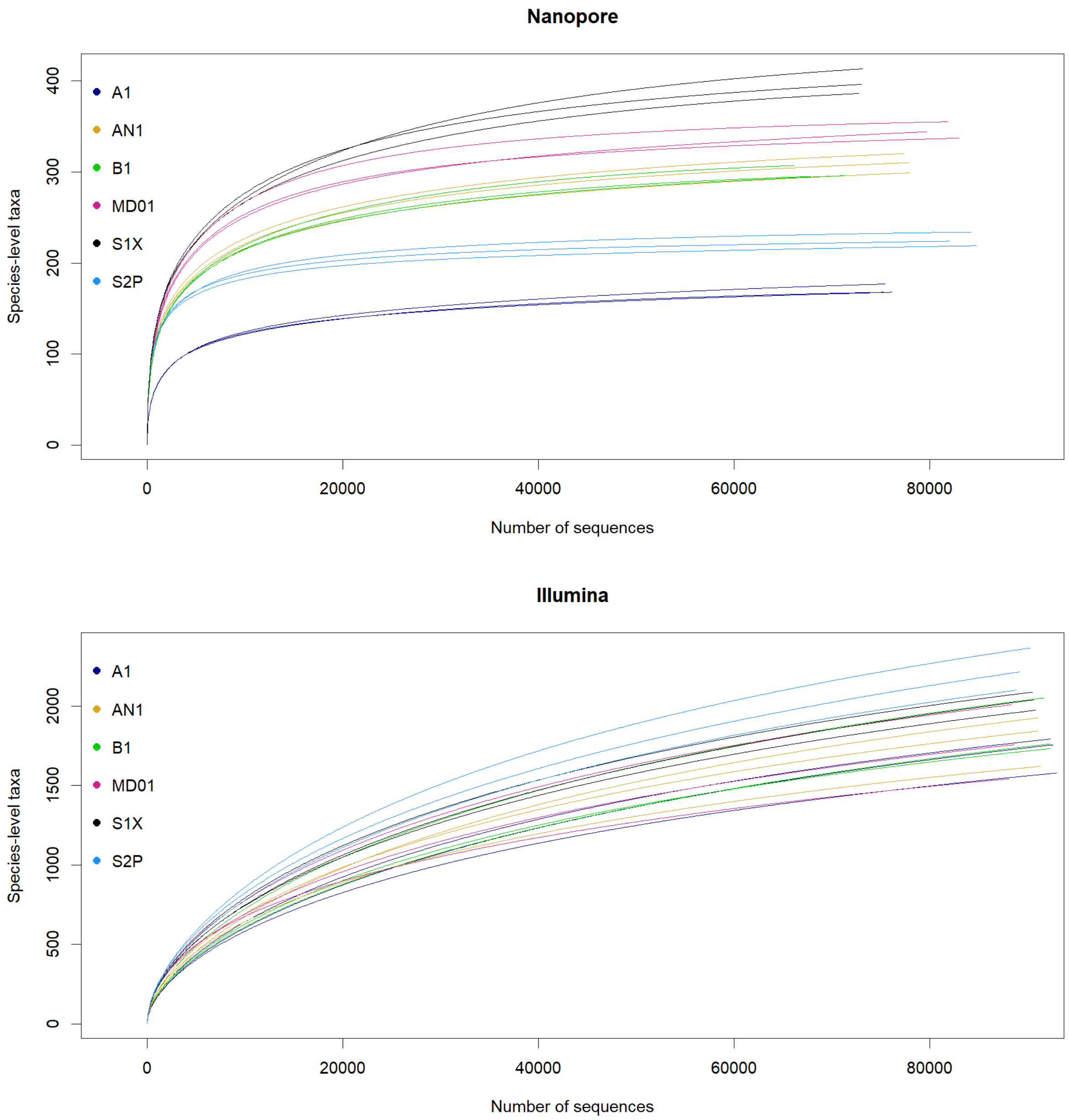

Figure S1: Rarefaction curves.

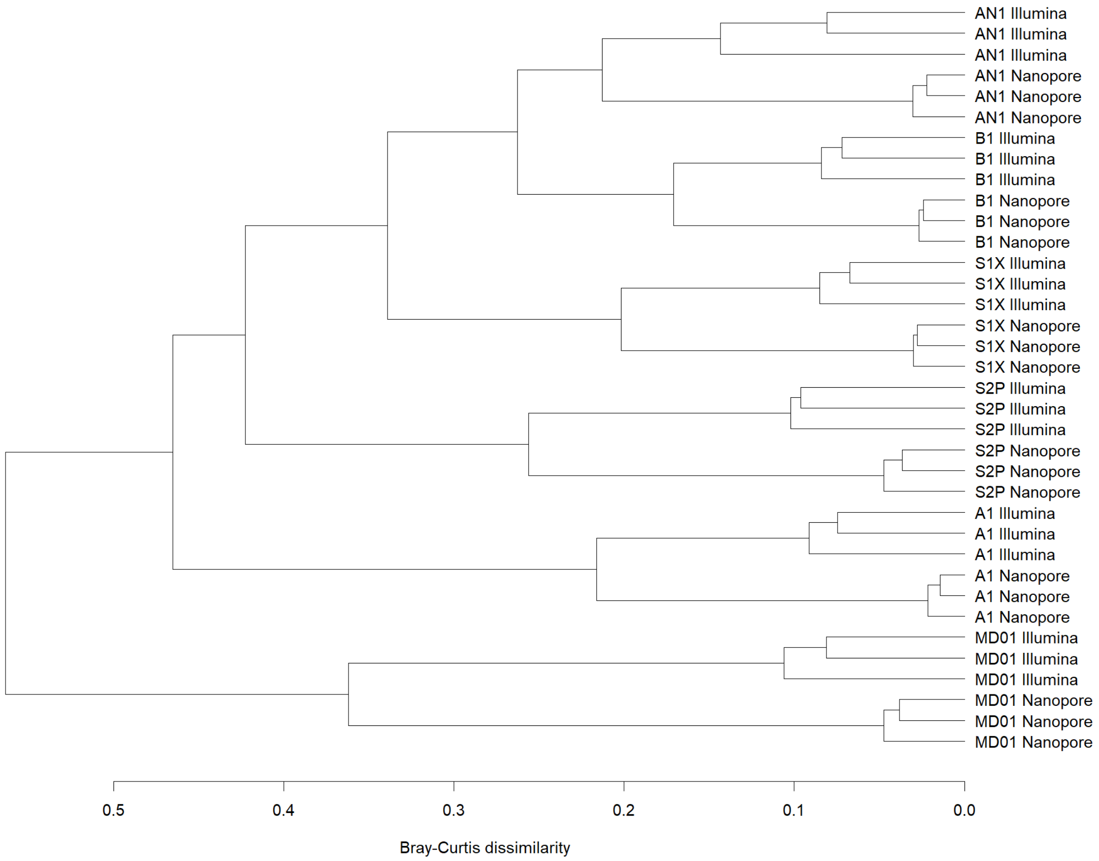

Figure S2: Clustering of the faecal samples based on their genus-level bacterial community structure

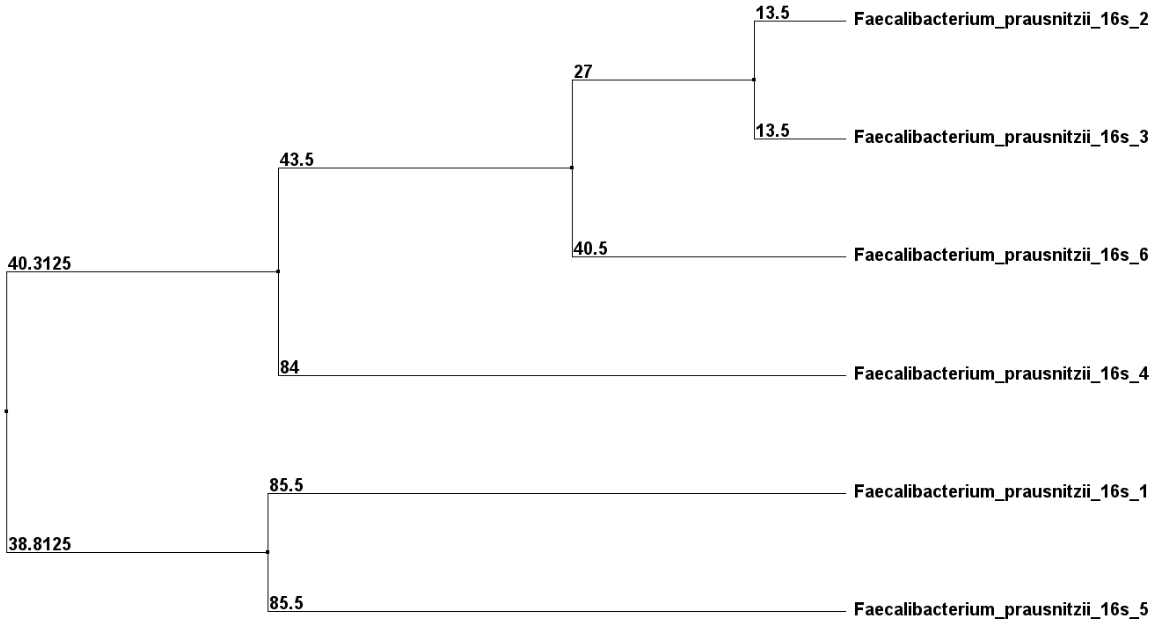

Figure S3: Average distance clustering of the 16S rRNA gene sequences of the *Faecalibacterium prausnitzii* AP34BHI genome after Clustal WS alignment
